# Author Correction: The Henna pigment Lawsone activates the Aryl Hydrocarbon Receptor and impacts skin homeostasis

**DOI:** 10.1038/s41598-020-65510-2

**Published:** 2020-05-20

**Authors:** Laura Lozza, Pedro Moura-Alves, Teresa Domaszewska, Carolina Lage Crespo, Ioana Streata, Annika Kreuchwig, Andreas Puyskens, Marina Bechtle, Marion Klemm, Ulrike Zedler, Bogdan Silviu Ungureanu, Ute Guhlich-Bornhof, Anne-Britta Koehler, Manuela Stäber, Hans-Joachim Mollenkopf, Robert Hurwitz, Jens Furkert, Gerd Krause, January Weiner, António Jacinto, Ioana Mihai, Maria Leite-de-Moraes, Frank Siebenhaar, Marcus Maurer, Stefan H. E. Kaufmann

**Affiliations:** 1 Department of Immunology, Max Planck Institute for Infection Biology, Charitéplatz 1, D-10117 Berlin, Germany; 2 Nuffield Department of Clinical Medicine, Ludwig Institute for Cancer Research, University of Oxford, Oxford, UK; 3 CEDOC, NOVA Medical School, NOVA University of Lisbon, Lisbon, 1169-056 Portugal; 4 Human Genomics Laboratory - University of Medicine and Pharmacy of Craiova, Craiova, Romania; 5 Leibniz-Forschungsinstitut fuer Molekulare Pharmakologie (FMP), Robert-Rössle-Strasse 10, 13125 Berlin, Germany; 6 Research Center of Gastroenterology and Hepatology, University of Medicine and Pharmacy of Craiova, Craiova, Romania; 7 Microarray Core Facility, Max Planck Institute for Infection Biology, Charitéplatz 1, D-10117, Berlin, Germany; 8 Biochemistry and Protein Purification Core Facility, Max Planck Institute for Infection Biology. Charitéplatz 1, D-10117 Berlin, Germany; 9 Laboratory of Immunoregulation and Immunopathology, INEM (Institut Necker-Enfants Malades), CNRS UMR8253, INSERM UMR1151 and Paris Descartes University, Paris, France; 10 Department of Dermatology and Allergy, Charité-Universitätsmedizin Berlin, Berlin, Germany; 11 Hagler Institute for Advanced Study, Texas A&M University, College Station, TX USA

Correction to: *Scientific Reports* 10.1038/s41598-019-47350-x, published online 26 July 2019

Figure 2F contains errors. The panel labelled as ‘Lawsone, 5 days, 10 μM’ was inadvertently duplicated from the ‘DMSO, 10 days, 10 μM’. The correct Figure 2 appears below as Figure 1.

**Figure 1:**
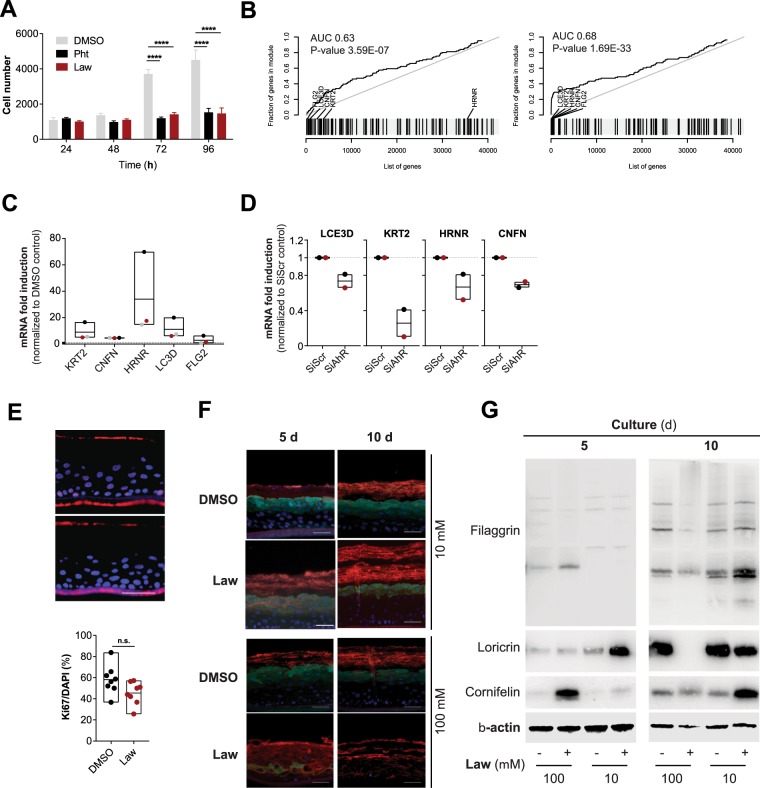
.

