# Supplementary material for: The Henna pigment Lawsone activates the Aryl Hydrocarbon Receptor and impacts skin homeostasis
Source: Sci Rep. 2019 Jul 26;9:10878. doi: 10.1038/s41598-019-47350-x (PMC6659674; doi:10.1038/s41598-019-47350-x)
Supplement: Supplementary file 1 — Supplementary Information [file 41598_2019_47350_MOESM1_ESM.pdf]

## Supplementary Information

# The Henna pigment Lawsone activates the Aryl Hydrocarbon Receptor and impacts skin homeostasis

Laura Lozza, Pedro Moura-Alves, Teresa Domaszewska, Carolina Lage Crespo, Ioana Streata, Annika Kreuchwig, Andreas Puyskens, Marina Bechtle , Marion Klemm, Ulrike Zedler, Silviu Ungureanu Bogdan , Ute Guhlich-Bornhof , Anne-Britta Koehler, Manuela Stäber, Hans-Joachim Mollenkopf, Robert Hurwitz, Jens Furkert, Gerd Krause, January Weiner 3rd, António Jacinto, Ioana Mihai, Maria Leite-de-Moraes, Frank Siebenhaar, Marcus Maurer, Stefan H.E. Kaufmann.

**Movie Supplement 1.** Effects of Lawsone expression on myeloid cell migration to a tail fin wound. Wounding response in 3dpf mpeg.mCherryCAAX SH378 mpx:GFP i114 line treated with Lawsone (10 μM), or DMSO. GFP (magenta) and bright field images were obtained every 1 min with a spinning-disk confocal microscope. The wound is located on the right side of the movie. Frame rate: 10 fps.

**Supplementary Dataset File 1-** Genes differently regulated on human keratinocytes after stimulation with Lawsone for 4 and 24 hours compared to Pam2CSK4 stimulation. Both conditions are normalized to keratinocytes stimulated with the vehicle control (DMSO).

**Table Supplement 1** - List of human Taqman primers, short interfering RNAs and zebrafish primers.

| **Human Taqman probes** | |
|---|---|
| Gene Symbol | Assay ID |
| AHR | Hs00169233_m1 |
| AHRR | Hs01005075_m1 |
| CNFN | Hs00261196_m1 |
| CYP1A1 | Hs01054797_g1 |
| FLG2 | Hs00418578_m1 |
| GAPDH | Hs02758991_g1 |
| HRNR | Hs02340614_m1 |
| KRT2 | Hs00166294_m1 |
| LCE3D | Hs00754375_s1 |
| **Short interfering RNAs** | |
| siRNA | Code |
| ON-TARGET plus Human AHR (196) siRNA, SMART-pool | L-004990-00-0005 |
| ON-TARGET plus Nono_targeting Pool siRNA | D-001810-10-05 |
| **Zebrafish primers** | |
| oligoname | sequence |
| zfCYP1A_qRT_Fw | GCATTACGATACGTTCGATAAGGAC |
| zfCYP1A_qRT_Rw | GCTCCGAATAGGTCATTGACGAT |
| zfBActin1_qRT_Fw | CGAGCAGGAGATGGGAACC |
| zfBActin1_qRT_Rw | CAACGGAAACGCTCATTGC |
| zfAhRRb_qRT_Fw1 | GACTACCTGGGATTTCATCAGACG |
| zfAhRRb_qRT_Rw1 | GAGCCGTCACAACATCCTCATC |
| zfAhRRa_qRT_Fw1 | GCCGCTGGCATATAACATGAGC |
| zfAhRRa_qRT_Rw1 | TGACGCTGTGTTCACGTCACTG |

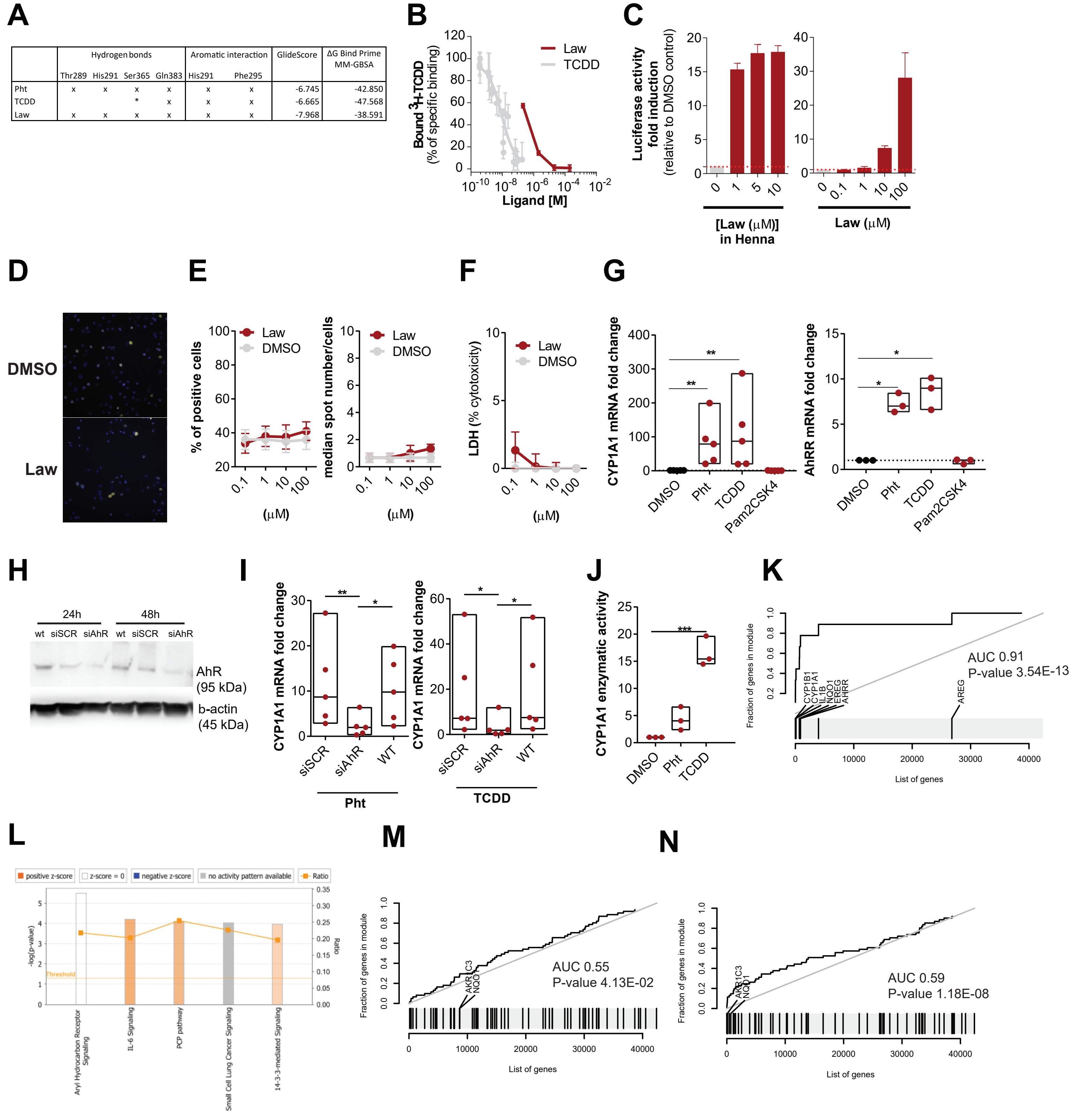


| | Hydrogen bonds | | | | Aromatic interaction | | GlideScore | ΔG Bind Prime MM-GBSA |
|---|---|---|---|---|---|---|---|---|
| | Thr289 | His291 | Ser365 | Gln383 | His291 | Phe295 | | |
| Pht | x | x | x | x | x | x | -6.745 | -42.850 |
| TCDD | | | * | x | x | x | -6.665 | -47.568 |
| Law | x | x | x | x | x | x | -7.968 | -38.591 |

**Figure Supplement 1. Lawsone activates AhR**

**(A)** Results for best scoring complexes for the hAhR homology model with bound Pht, TCDD and Lawsone including potential hydrogen bonds, aromatic interactions, as well as binding affinity estimation by GlideScore and ΔG bind. **(B)** Binding of Lawsone to AhR. **(C)** Luciferase activity of AhR-reporter THP1 cells stimulated for 4 hours (h) with Henna or Lawsone (Law). **(D)** Representative staining of H2A.X histone phosphorylation in nuclei of human primary keratinocytes (HEK) cells after exposure to DMSO or Lawsone (10 µM). **(E, left)** Percentage of positive H2A.X cells and **(E, right)** H2A.X spot number/cell after 4h exposure to different concentrations of Lawsone (red) or DMSO control (grey). **(F)** Lactate dehydrogenase (LDH) released by HEK cells stimulated for 24h with different concentrations of Lawsone (red), or DMSO control (grey). **(G)** CYP1A1 and AHRR expression after 4h Phthiocol (Pht, 50 µM), TCDD or Pam2CSK4 (0.236 µM) stimulation of HEK cells normalized to DMSO (each dot represents one individual). **(H)** HEK cells were transfected with AhR-siRNA (siAhR) or Scramble control (siScr). Western Blot analysis of AhR on HEK cells after AhR silencing by RNAi (siAhR) compared to WT and scramble control (siScr) at 24 and 48 h. One representative experimet is shown. **(I)** CYP1A1 expression after 4h stimulation with Pht (50 µM) or TCDD (10 nM) normalized to DMSO. **(J)** CYP1A1 enzymatic activity in HEK cells treated with Pht (50 µM) or TCDD (10 nM) compared DMSO for 48h. **(K)** AhR-target gene enrichment after 24h Lawsone stimulation (10 µM) relative to TLR2 stimulation (Pam2CSK4, 0.236 µM).**(L)** Ingenuity pathway analysis of Top canonical pathways differentially regulated upon 4h Lawsone (10 µM) stimulation of HEK cells, when compared to DMSO. **(M, N)** Nrf2-target gene enrichment at **(M)** 4h and **(N)** 24h of treatment as described in J. Area under the curve (AUC), q-value and highly enriched genes are indicated. **(C, E, F, G and I)** Data from at least 3 independent experiments are shown, **(C, E, F)** Mean+ S.E.M. (G-I) Floating Bars, Mean Min to Max. **(E, F)** Two-way ANOVA with Fisher's test, **(G, H and I)** One-way ANOVA with Fisher's test. *P<0.05; **P<0.01; ***P<0.001.

**Figure Supplement 1**

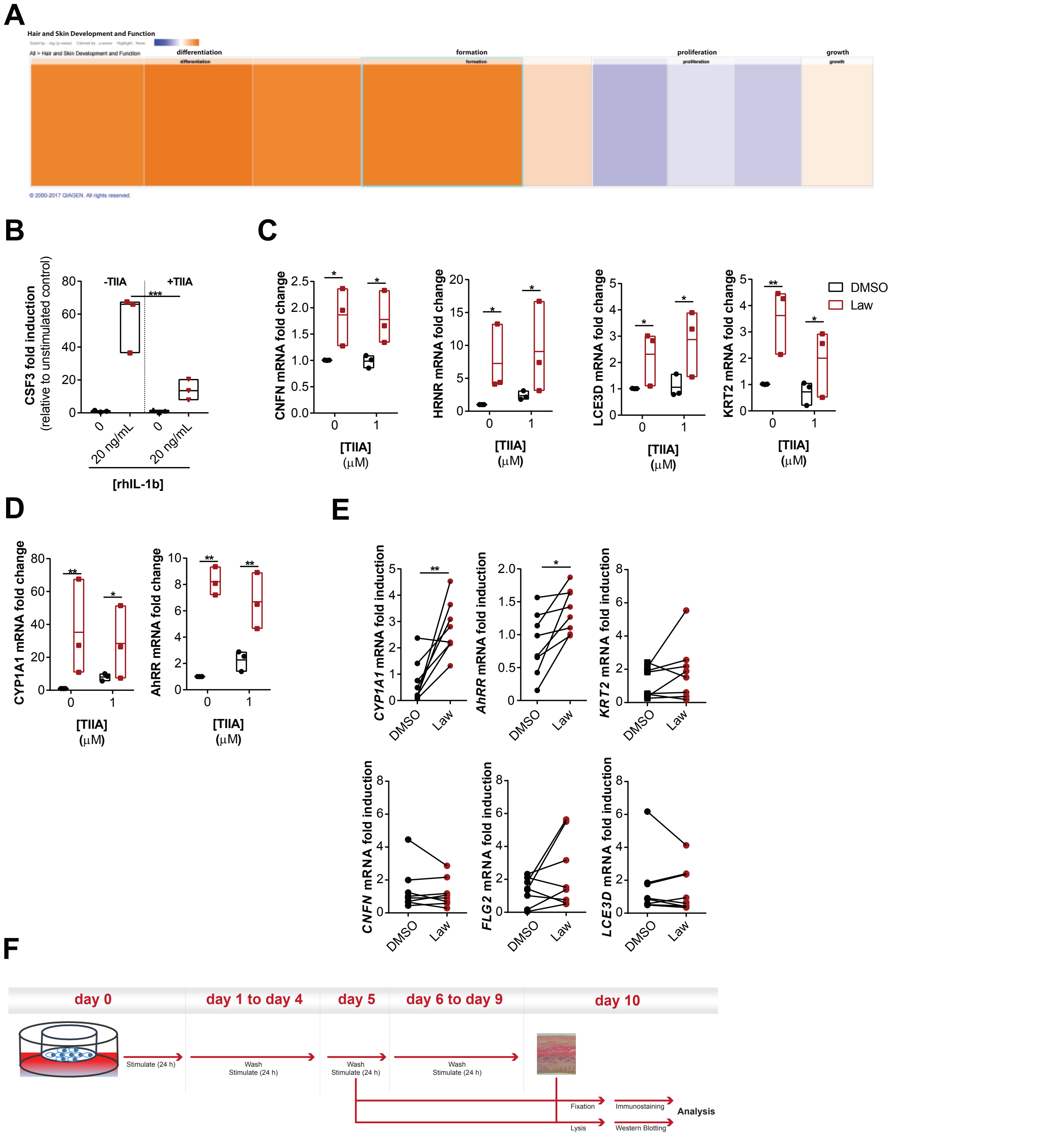


**Figure Supplement 2. Lawsone modulates keratinocyte proliferation and differentiation**
**(A)** Ingenuity pathway analysis of predicted hair and skin development/functions differentially regulated upon 4h Lawsone (10 μM) stimulation of HEK cells compared to DMSO. Induction is depicted in orange and inhibition in blue. **(B-D)** HEK cells were treated with DMSO or 1 μM of Tanshinone IIA (TIIA) for 15 min followed by stimulation with **(B)** recombinant human IL-1β (rhIL-1β, red) and **(C, D)** Lawsone (red) or DMSO (black) for 24h. **(B)** CSF3 expression upon stimulation in the presence or absence of TIIA. **(C, D)** Epidermal differentiation complex **(C)** or AhR dependent **(D)** gene expression after Lawsone (10 μM) stimulation. **(E)** CYP1A1, AHRR, KRT2, CNFN, FLG2 and LCE3D expression in cells from total biopsies (1 cm2) of human skin treated for 24h with Lawsone (10 μM) or vehicle DMSO control. HRNR was not detected. Fold induction is calculated as 2-ΔΔCt relative to the mean (ΔΔCt) of all samples. **(F)** Layout of human skin equivalent experiments performed. **(B)** 1 representative experiment out of 3 experiments is depicted, **(C, D)** Pooled data from 3 independent experiments are shown. **(E)** Data of 8 different individuals are shown, **(B-D)** Floating bars, Mean Min to Max. Two-way ANOVA with Fisher's test. **(E)** Paired Student's t-test. *$P<0.05$; **$P<0.01$, ***$P<0.001$.

**Figure Supplement 2**

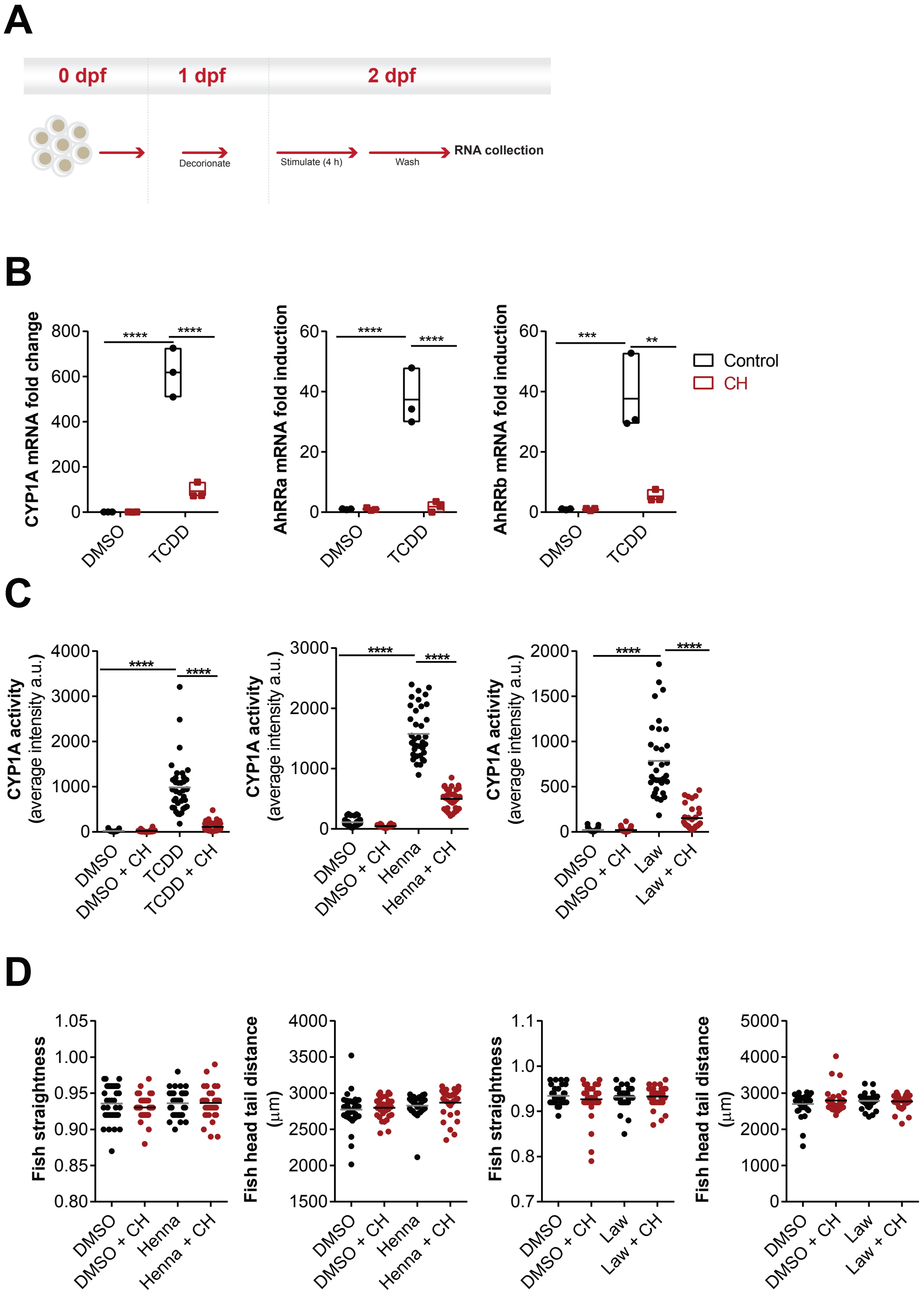


**Figure Supplement 3. AhR activation in zebrafish larvae**

**(A)** Scheme of zebrafish larvae stimulation experiments. **(B)** Fold induction of CYP1A, AhRRa and AhRRb transcripts from zebrafish larvae (2 days post-fertilization, dpf) treated (red squares) or not (black circles) for 2h with 5 μM of CH223191 (CH), followed by further 4h stimulation with TCDD (10 nM) or DMSO vehicle control. Triplicates of 12 larvae depicted in each data point. **(C)** CYP1A enzymatic activity expressed as average intensity of resorufin detection per larva after 4h stimulation with TCDD (10 nM), Henna (equivalent to 10 μM Lawsone) or Lawsone (10 μM) for 4h, in the presence or absence of CH (5 μM). Each dot represents one larva. **(D)** Analysis of fish straightness and head to tail distance as a readout of toxicity in 2dpf zebrafish larvae exposed for 4h with Henna (equivalent to 10 μM Lawsone), Lawsone (10 μM) or DMSO, in the presence (red dots) or absence (black dots) of CH223191 (5 μM). **(B)** 1 representative experiment out of 3 is shown, **(C, D)** 1 representative experiment out of 3 is shown (n=36 larvae per condition). **(B)** Floating bars, Mean Min to Max. (B) Two-way ANOVA with Bonferroni's test. **(C, D)** Two-way ANOVA with Fisher's test. **P<0.01; ***P<0.001; ****P<0.0001.

**Figure Supplement 3**

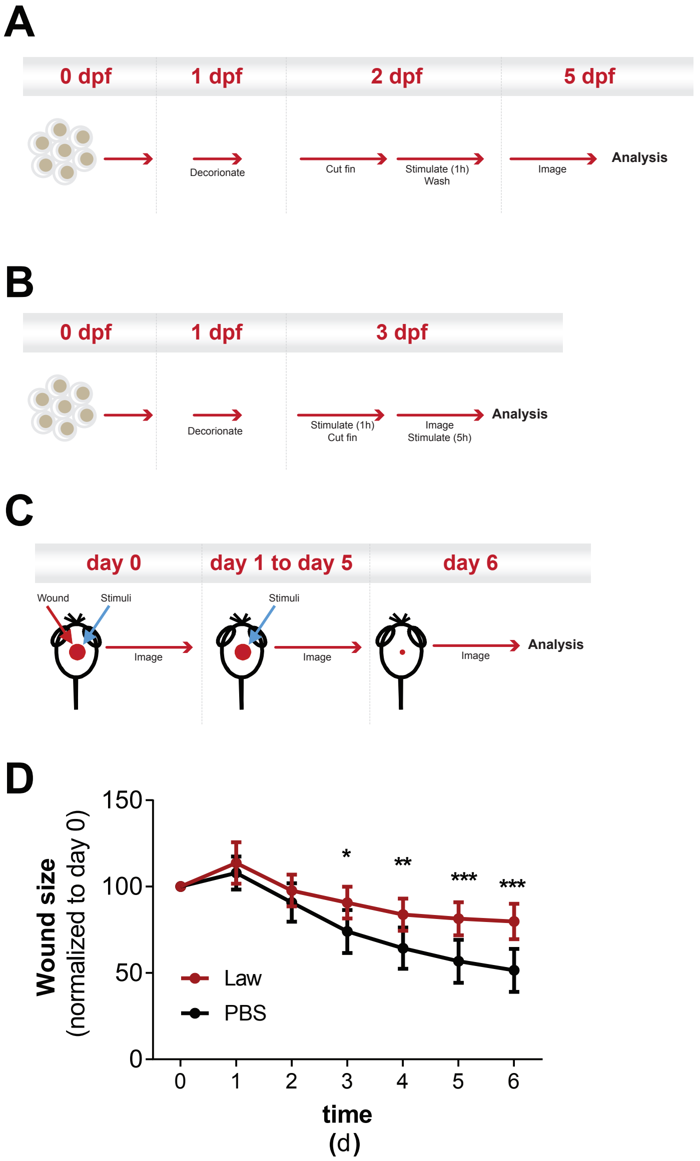


**Figure Supplement 4. Wound healing, regeneration and human skin equivalent experiments**
**(A)** Experimental layouts of: **(A)** zebrafish larvae fin regeneration assay, **(B)** zebrafish larvae wound healing and neutrophil dynamics assay and **(C)** mouse wound healing model. **(D)** Wound healing after local treatment with Lawsone (10 μM) or PBS on excisions of C57BL/6 mice (8 mice/group). Data from 2 pooled experiments, Mean+ S.E.M. Two-way ANOVA with Fisher's test. *P<0.05; **P<0.01; ***P<0.001.
